# Emerging Role of Epitranscriptomics in Diabetes Mellitus and Its Complications

**DOI:** 10.3389/fendo.2022.907060

**Published:** 2022-05-27

**Authors:** Xinqian Geng, Zheng Li, Ying Yang

**Affiliations:** ^1^ Department of Endocrinology, The Affiliated Hospital of Yunnan University and the Second People’s Hospital of Yunnan Province, Kunming, China; ^2^ School of Pharmacy, Guangdong Pharmaceutical University, Guangzhou, China

**Keywords:** epitranscriptomics, diabetes, diabetic cardiomyopathy, diabetic nephropathy, diabetic retinopathy, diabetic wounds, diabetic neuropathy

## Abstract

Diabetes mellitus (DM) and its related complications are among the leading causes of disability and mortality worldwide. Substantial studies have explored epigenetic regulation that is involved in the modifications of DNA and proteins, but RNA modifications in diabetes are still poorly investigated. In recent years, posttranscriptional epigenetic modification of RNA (the so-called ‘epitranscriptome’) has emerged as an interesting field of research. Numerous modifications, mainly *N^6^*-methyladenosine (m^6^A), have been identified in nearly all types of RNAs and have been demonstrated to have an indispensable effect in a variety of human diseases, such as cancer, obesity, and diabetes. Therefore, it is particularly important to understand the molecular basis of RNA modifications, which might provide a new perspective for the pathogenesis of diabetes mellitus and the discovery of new therapeutic targets. In this review, we aim to summarize the recent progress in the epitranscriptomics involved in diabetes and diabetes-related complications. We hope to provide some insights for enriching the understanding of the epitranscriptomic regulatory mechanisms of this disease as well as the development of novel therapeutic targets for future clinical benefit.

## Introduction

Diabetes mellitus (DM) is a complex metabolic disorder that is characterized by increased blood glucose concentrations; it has reached epidemic proportions worldwide (1). It is well established that high levels of glucose contribute to higher risks of microvascular and macrovascular diseases, including diabetic cardiomyopathy, retinopathy, nephropathy, neuropathy, and ulcers, which are major causes of disability and mortality in patients with diabetes (2–6). The global diabetes prevalence in adults aged 20-79 was estimated to be 10.5% (536.6 million people) in 2021, and it is estimated to rise to 12.2% (783.2 million) in 2045. Thus, it contributes to increasingly high health care costs (1). Diabetes-related health care expenditure was 966 billion USD in 2021 and is projected to reach 1,054 billion USD by 2045. This would represent an increase of 9.1% compared to that in 2021 (1). Substantial evidence has demonstrated that epigenetic regulatory mechanisms (e.g., histone modifications, DNA methylation and noncoding RNA) play a pivotal role in the occurrence and development of diabetes (7–9). However, the identification of posttranscriptional epigenetic modification of RNA (epitranscriptomic modification) in diabetes and its complications is still in its nascent stage.

Similar to DNA methylation modification, cellular RNA is also modified with diverse chemical modifications, such as *N^6^*-methyladenosine (m^6^A), 5-methylcytidine (m^5^C), inosine (I), and pseudouridine (Ψ) (10, 11). More than 160 types of chemical modifications have been discovered in RNAs to date (11). Such modifications can influence the metabolism and function of RNAs and have been regarded as key regulators of gene expression (12). The multitude of modifications in RNAs lead to an emerging scientific field of “RNA epigenetics” or “epitranscriptomics”, which could add a new layer of control of genetic information (11, 13–16). It is believed that epitranscriptomics, which are reversible modifications of RNAs, play a crucial role in various disease conditions, such as cancer, obesity, leukemia, neurological disorders and diabetes (17–20). For instance, methyltransferase-like 3 (Mettl3)-mediated m^6^A modification was reported to be connected to hepatogenous diabetes in mice fed a high-fat diet (HFD) (20).

In consideration of the influence on human health, abundant work has focused on understanding the epigenetic mechanisms involved in the pathophysiology of diabetes mellitus and how these processes are disrupted in the diabetic state. In this review, we provide a brief overview of epitranscriptomics and summarize the recent progresses in the research of epitranscriptomics involved in diabetes and diabetes-related complications, which may provide novel insights for understanding the pathogenesis of diabetes and indicate promising therapeutic targets for this disease.

## Brief Overview of RNA Modifications

**Figure 1:**
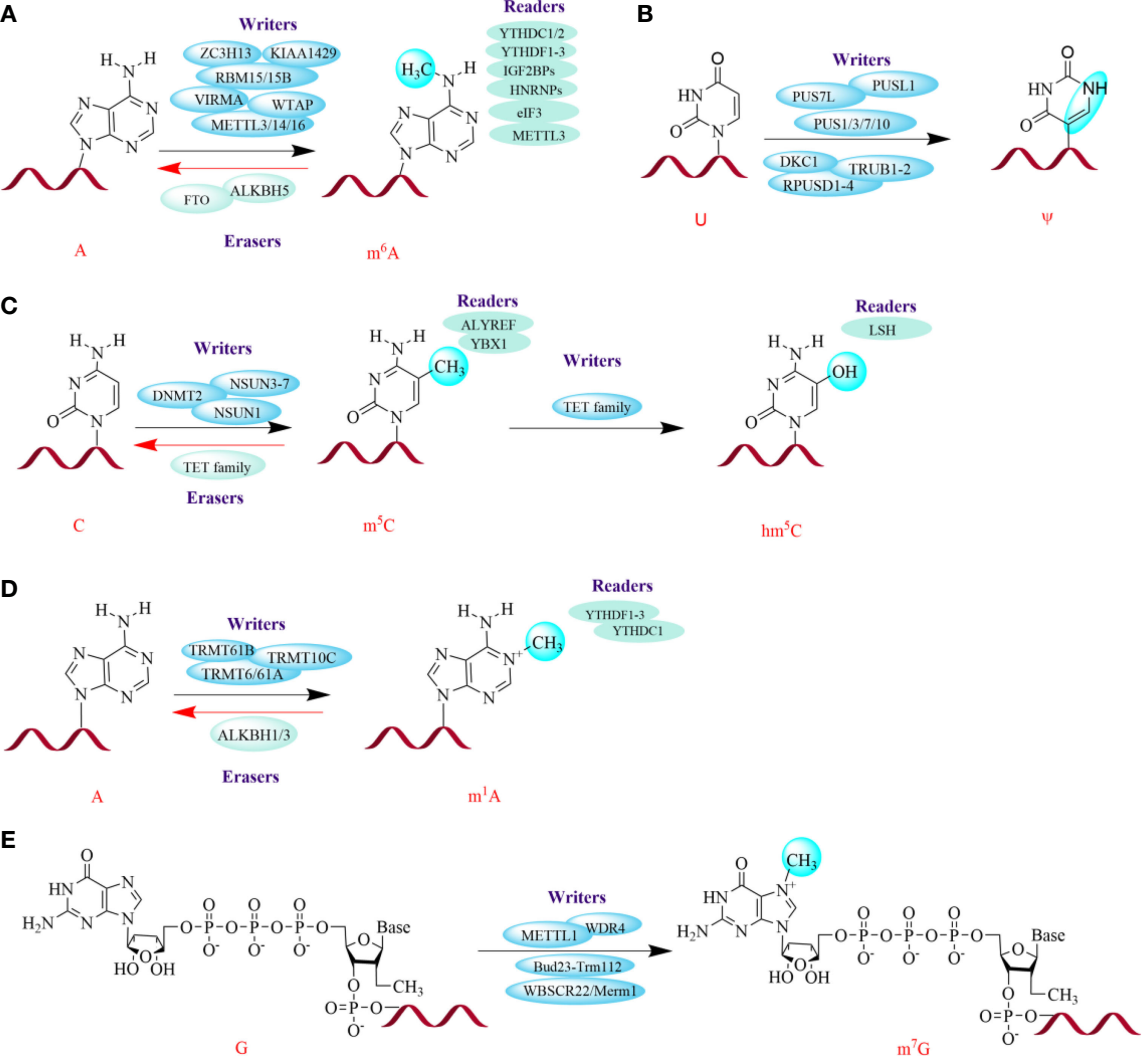
Several major chemical modifications in RNAs. The green spheres on the bases represent modification sites. The red helices represent RNA. The dynamic and reversible modifications are mediated by three groups of proteins: writers (install modifications on specific sites), erasers (remove modifications), and readers (recognize modifications). *METTL3/14/16*, Methyltransferase-like 3, 14, 16; *WTAP*, Wilms' tumor 1-associating protein; *RBM15/15B*, RNA-binding motif protein 15/15B; *VIRMA*, Vir-like m6A methyltransferase associated; *ZC3H13*, Zinc finger CCCH domain-containing protein 13; *FTO*, Fat mass and obesity-associated protein; *ALKBH5*, ALKB homolog 5; *YTHDF1/2/3*, YT521-B homology (YTH) protein families including YTH domain family 1, 2, 3; *YTHDC1/2*, YTH domain containing 1, 2; *IGF2BPs*, Insulin-like growth factor 2 mRNA-binding proteins; *HNRNPs*, Heterogeneous nuclear ribonucleoproteins; *eIF3*, Eukaryotic translation initiation factor; *TRMT6/61A*, tRNA methyltransferase 6, 61A; *PUSs*, Pseudouridine synthases; *PUS7L*, PUS7-like; *PUSL1*, PUS-like 1; *DKC1*, Dyskerin PUS1; *RPUSD1/2/3/4*, RNA PSU domain-covering 1, 2, 3, 4; *TRUB1/2*, TruB PSU class member 1, 2; *NSUN3-7*, NOL1/NOP2/SUN domain (NSUN) family of proteins 3, 4, 5, 6, 7; *DNMT2*, DNA methyltransferase (DNMT) homolog-2; *TET family*, Ten-eleven translocation family enzymes; *ALYREF*, Aly/REF export factor; *YBX1*, Y box binding protein 1; *LSH*, lymphoid specific helicase; *WBSCR22*, Williams-Beuren syndrome chromosomal region 22 protein, also called BUD23; *Merm1*, Metastasis-related methyltransferase 1; *WDR4*, WD 159 repeat domain 4.

Many studies have reported that chemical modifications of RNA transcripts are also key regulators of gene expression that participate in RNA splicing, transcript stability, translation, nucleation, and RNA–protein interactions (12, 18, 21–26). Modifications on the 5’ cap and 3’ poly(A) tail of mRNA play crucial roles in mRNA metabolism; their roles have been extensively reviewed elsewhere (4). Thus, in the following sections, we briefly summarize several internal chemical modifications of coding and noncoding RNA (**Figure 1**). For a detailed discussion on this topic, readers can refer to several excellent reviews (10, 11, 13, 15, 27).

### *N^6^*-Methyladenosine (m^6^A)

m^6^A is the most abundant and prevalent internal mRNA modification in eukaryotic mRNA and is a critical posttranscriptional mRNA regulator (26, 28, 29). m^6^A is a stable and conserved chemical derivative of adenosine that accounts for more than 60% of all RNA modifications (26). It was initially identified in the 1970s in mRNA, and later reported to exist in non-coding RNAs, including lncRNA, rRNA, and circRNAs (20–23). The dynamic and reversible m^6^A patterns are mediated by three groups of proteins: writers, erasers, and readers (26, 30). m^6^A methyltransferase complexes (MTCs), termed “writers”, can selectively install m^6^A-modified sites upstream of information processing; these writers mainly include methyltransferase-like 3/14 (METTL3/14) and Wilms’ tumor 1-associating protein (WTAP) and KIAA1429 (31–33). Additionally, there are a handful of regulatory factors that contribute to the activity and specificity of the catalytic complex; these include METTL16, RNA-binding motif protein 15/15B (RBM15/15B), vir-like m^6^A methyltransferase associated (VIRMA), and zinc finger CCCH domain-containing protein 13 (ZC3H13) (34–37). The demethylases, also known as “erasers”, are mainly composed of fat mass and obesity-associated protein (FTO) and ALKB homolog 5 (ALKBH5) (38, 39). However, the role for FTO as an m^6^A eraser is controversial since several studies have showed that FTO preferentially demethylates *N^6^*,2’-O-dimethyladenosine (m^6^Am) rather than m^6^A (40–43). Further studies showed that FTO might target at nuclear RNA over mRNA; this was demonstrated by the phenomena that more RNA methylation accumulated in snRNAs and small nucleolar RNAs (snoRNAs) in *Fto -* knockout cells compared with mRNA (40, 41). Moreover, m^6^A can be recognized by m^6^A-binding proteins, also called “readers”; this group principally consists of YT521-B homology (YTH) protein families, including YTH domain family 1/2/3 (YTHDF1/2/3) and YTH domain containing 1/2 (YTHDC1/2), insulin-like growth Factor 2 mRNA-binding proteins (IGF2BPs), heterogeneous nuclear ribonucleoproteins (HNRNPs), eukaryotic translation initiation factor (eIF3), and METTL3 (44–50).

### *N^1^*-Methyladenosine (m^1^A)

m^1^A methylation occurs at the *N^1^* position of adenosine and accumulates in tRNA, rRNA and mRNA (13, 44, 51). In tRNA and rRNA, m^1^A maintains the tertiary structure by affecting translation (52, 53). In mRNA, m^1^A is primarily located in highly structured or GC-rich regions of 5’ untranslated regions (UTRs), although it is also found in the coding sequence (CDS) and 3’UTR (54). Similar to mRNA writers, tRNA methyltransferase 6/61A (TRMT6/61A), TRMT61B, and TRMT10C could contribute to m^1^A methylation in tRNA. Additionally, these tRNA methyltransferases can also regulate m^1^A methylation in mRNAs (55). Interestingly, YTHDF1-3 and YTHDC1 can also act as “readers” in m^1^A modifications by binding to m^1^A-bearing RNA (56). Subsequently, this modification can be demethylated by ALKBH1 and ALKBH3, which function as erasers (54, 57).

### 5-Methylcytosine (m^5^C)

Methylation at the 5 position of cytosine (m^5^C) was also first discovered to be gathered at the UTRs of mRNA, especially in GC-rich regions, which is similar to that of m^1^A (58–60). Subsequently, the NOL1/NOP2/SUN domain (NSUN) family of proteins (NSUN1, NSUN3/4/5/6/7) and DNA methyltransferase (DNMT) homolog-2 (DNMT2) were reported to serve as “writers” in tRNAs and rRNAs, but only NSUN2 can fix m^5^C on mRNA (27, 61–63). Later, a specific mRNA m^5^C-binding protein, Aly/REF export factor (ALYREF), was reported as a “reader” of m^5^C (59). Recently, Chen et al. found that YBX1 acts as another m^5^C reader that can maintain the stability of target mRNAs in bladder cancer (64). However, little is known about the “eraser” protein(s) for this modification.

### 5-Hydroxymethylcytosine (hm^5^C)

hm^5^C has been identified as a transformed form of m^5^C (13, 27). Similar to that in DNA, m^5^C in RNA can also be catalyzed into hm^5^C by ten-eleven translocation (TET) family enzymes (65, 66). Furthermore, methylated RNA immunoprecipitation sequencing (called MeRIP-seq) in *Drosophila* S2 cells illustrated that Tet-family enzymes tend to act on coding regions, thus promoting mRNA translation (67). In tRNAs, several studies have revealed that ALKBH1 is also involved in the biogenesis of hm^5^C and 5-formyl-2′-*O*-methylcytidine (f^5^Cm) (68, 69). Interestingly, a chromatin remodeling factor, lymphoid specific helicase (LSH), is considered a reader of hm^5^C in cancer. It has been reported that LSH can interact with TET2. This increases the expression of the latter and favors genome stability by silencing satellite expression by improving the levels of hm^5^C in metastasis (70).

### *N^7^*-Methylguanosine (m^7^G)

m^7^G is the most ubiquitous RNA cap modification on the 5’ end of eukaryotic mRNAs and exists in mRNA, tRNA and rRNA (55, 71, 72). In humans, the 1639 site of 18S rRNA is m^7^G-modified by the Williams-Beuren syndrome chromosomal region 22 protein (WBSCR22, also called BUD23)/metastasis-related methyltransferase 1 (Merm1), whereas in yeast, the Bud23-Trm112 heterodimer is required for *N^7^*-methylation of G_1575_ in the small ribosomal subunit rRNA (73, 74). Similar to m^7^G modification in rRNA, m^7^G tRNA modification is catalyzed by METTL1 and WD repeat domain 4 (WDR4) in humans and the heterodimeric complex of Trm8p/Trm82p in yeast (75–77). Moreover, METTL1/WDR4 also act as “writers” for m^7^G modification on mRNA (72).

### Pseudouridine (Ψ)

Ψ is the isomerization of uridine. It is the most abundant modification in cellular RNA and appears in various RNAs, including small nucleolar RNA, snRNA, and mRNA, especially in rRNA and tRNA (13, 78). This modification can be dynamically regulated by “writers”, pseudouridine synthases (PUSs), and 13 synthases have been discovered to date, including PUS7-like (PUS7L), PUS-like 1 (PUSL1), dyskerin PUS1 (DKC1), RNA PSU domain-covering 1/2/3/4 (RPUSD1/2/3/4), TruB PSU class member 1/2 (TRUB1/2), and PUS1/3/7/10 (79, 80). Notably, although Ψ can improve translation, it can negatively affect protein expression (27, 81).

### RNA Editing

Adenosine-to-inosine RNA editing (A-to-I editing), also termed I, is a type of programmed alteration that has broad physiologic significance (27, 82). It has been demonstrated that A-to-I editing can be regulated by a family of adenosine deaminases acting on RNA (ADARs) (82–84). Although three ADAR genes have been found in mammals, only ADAR1 and ADAR2 possess deaminase activity in regions of RNA with double-stranded character (82). Since A-to-I editing can fasten pairs of nucleotides, this modification can affect the secondary structure of mRNA in metazoans and further influence RNA structure stability (85).

Cytidine-to-uridine RNA editing (C-to-U editing), namely, U, is the other kind of canonical but rare RNA editing in humans (86, 87). Previous studies have reported that U prefers to be located in 3’ UTRs and is catalyzed by apolipoprotein B-editing enzyme catalytic polypeptide-1 (APOBEC1) (88, 89). Subsequently, in a study of small intestine and liver mRNA in mice, the C-to-U editing of RNA was demonstrated to be associated with the protein level, although the exact relationship between this modification and the expression of transcripts is unknown (86).

## Methods for Detecting RNA Modifications

Recent years, the development of new technologies dramatically promoted the understanding of functions of RNA modifications and the discovery of new modifications. Meanwhile, the increasing interest in epitranscriptomics has also prompted the progresses in developing new detecting tools. It is recognized that total level of m^6^A modification in RNA can be detected by liquid chromatography-tandem mass spectrometry (LC-MS/MS) and colorimetric method (90, 91). Many high-throughput sequencing methods have been developed to detected m^6^A modification based on different mechanisms, including antibody-dependent single-nucleotide resolution sequencing (PA-m^6^A-seq, m^6^A-CLIP and miCLIP), chemical labeling sequencing (m^6^A-label-seq and m^6^A-SEAL), MeRIP-m^6^A-seq (the most widely used method so far), endoribonuclease-based sequencing (m^6^A-REF-seq and MASTER-seq), and other methods (such as m^6^A-LAIC-seq, DART-seq and Nanopore-seq), endoribonuclease-based sequencing (m^6^A-REF-seq and MASTER-seq), and other methods (such as m^6^A-LAIC-seq, DART-seq and Nanopore-seq) (11, 92–96). Recently, an antibody-independent single-nucleotide-resolution method named m^6^A-SAC-seq has been developed (97). Similarly, m^1^A modification can be detected by LC-MS method as well as high-throughput sequencing strategies (including m^1^A-ID-seq, m^1^A-seq, m^1^A-IP-seq, m^1^A-seq-TGIRT, m^1^A-MAP, and m^1^A-quant-seq) (11, 98, 99). The level of m^5^C methylation can be detected by LC-MS, Bisulfite-seq and high-throughput sequencing methods (including m^5^C-RIP-seq, RNA-BisSeq, miCLIP-seq, TAWO-seq, Aza-IP-seq, and Nanopore-seq) (11, 99–102). LC-MS/MS method and MeRIP-seq are also available for the detection of hm^5^C and m^7^G modifications (11, 72, 103). Besides, other sequencing strategies, such as m^7^G-seq and miCLIP-seq, are used for quantitative detection of m^7^G modification (72, 104). As for Ψ site, it can not only be tested by Ψ-Seq, Pseudo-Seq, PSI-Seq, Nanopore-seq and CeU-Seq, but also be predicted by computational methods such as iRNA-PseU and XG-PseU (105, 106).

Although many detecting technologies have been developed, cautions should still be taken when detecting chemical modifications on RNAs by specific methods considering their limited sensitivity and accuracy. Moreover, to better understand the multiple detecting techniques of RNA modifications, readers can refer to several professional studies (11, 72, 98, 99, 106).

## The Role of Epitranscriptomics in Diabetes

Normal blood glucose levels are achieved by a feedback loop between pancreatic β cells producing insulin and insulin-sensitive tissues (primarily liver, adipose, and muscle) (50). DM is characterized by chronic hyperglycemia resulting from impaired insulin action (i.e., insulin resistance (IR) and/or secretion) (50). It is well established that both genetics and the environment play major roles in the development of diabetes (107). Among environmental factors, reversible RNA modifications have recently raised increasing interest from researchers and have been demonstrated to affect glucose homeostasis (20, 108, 109). As one of the most widespread RNA modifications, m^6^A was first reported to stimulate glucose oxidation in rat adipocytes in 1982; this finding implies that m^6^A could affect glucose metabolism in fat cells (109). Later, it was discovered that both the m^6^A level and *METTL3* expression were increased in the livers of patients with type 2 diabetes mellitus (T2DM), and their expression was positively associated with IR by promoting the expression of fatty acid synthase (*Fasn*) (110). Consistently, hepatocyte-specific knockout of *Mettl3* mice feeding with HFD ameliorated insulin sensitivity and reduced fatty acid synthesis by down-regulating the expressions of fatty acid synthesis and oxidation genes (such as *Fasn*, *Sirt1*, *Ehhadh*, and *Foxo1*) in a m^6^A-mediated way (110). Moreover, Li et al. also revealed that the overexpression of *Mettl3* aggravated IR in HFD-fed mice. RNA-seq and miCLIP-seq data showed that some candidate genes (such as *Lpin1*, *Lpin2*, *G6pc*, *Pck1*, and *Ppara*) were down-regulated in Mettl3^Ctrl^ mice feeding with HFD (20). Significantly, *lpin1* has been recognized as an important factor in lipid metabolism and IR (111, 112). It is demonstrated that silencing of *lipin-1* in C2C12 myotubes resulted in elevated ceramide accumulation, but attenuated insulin-induced Akt phosphorylation and glucose uptake, thereby leading to IR (113). Correspondingly, Li et al. further demonstrated that knockdown of *Mettl3* could stabilize *Lpin1* mRNA by regulating m^6^A levels, thus improving IR. These findings reveal a key role of *Mettl3*-mediated m^6^A modification in hepatogenous diabetes (20, 113). IGF2BP2, an m^6^A reader, has been identified as a T2DM-associated gene. Single nucleotide polymorphisms (SNPs) in the human IGF2 mRNA binding protein 2 (IMP2/IGF2BP2) are associated with increased risk of T2DM in different populations, such as Chinese and Indians (114, 115). Further study demonstrated that variants in *IMP2* locus exerted their effects in T2DM susceptibility predominantly by impairing insulin secretion rather than reducing insulin sensitivity (116–118). Moreover, a recent study showed that IMP2/IGF2BP2 working together with the IGF2-AKT-GSK3β-PDX1 pathway, could promote β-cell proliferation and enhance insulin secretion (119).

As the pivotal pathogenic mechanism of diabetes, β cell function has been associated with RNA modifications. It was shown that the deletion of *Mettl3* in islet β cells may diminish the modification of m^6^A and suppress the expression of insulin secretion-related genes, thereby inducing β cell failure and hyperglycemia (120). Notably, a recent study of islet cells from patients with T2DM further suggested that m^6^A was significantly reduced in β-cells and broadly located in insulin secretion-related genes. Moreover, further analyses revealed that a lower level of m^6^A resulting from knockdown of *Mettl3* or *Mettl14* could impair glucose-stimulated insulin secretion (GSIS) by inhibiting the insulin/IGF1–AKT–PDX1 pathway (121). For METTL14, specific knockout of *METTL14* in β-cells resulted in reduced insulin secretion and induced glucose intolerance by activating the IRE1α/sXBP-1 pathway (122). Collectively, it is obvious that METTL3/14 is vital for islet β-cell biology and glucose homeostasis. Thus, METTL3/14 might be potential therapeutic targets for the treatment of β-cell failure in diabetes.

**Figure 2:**
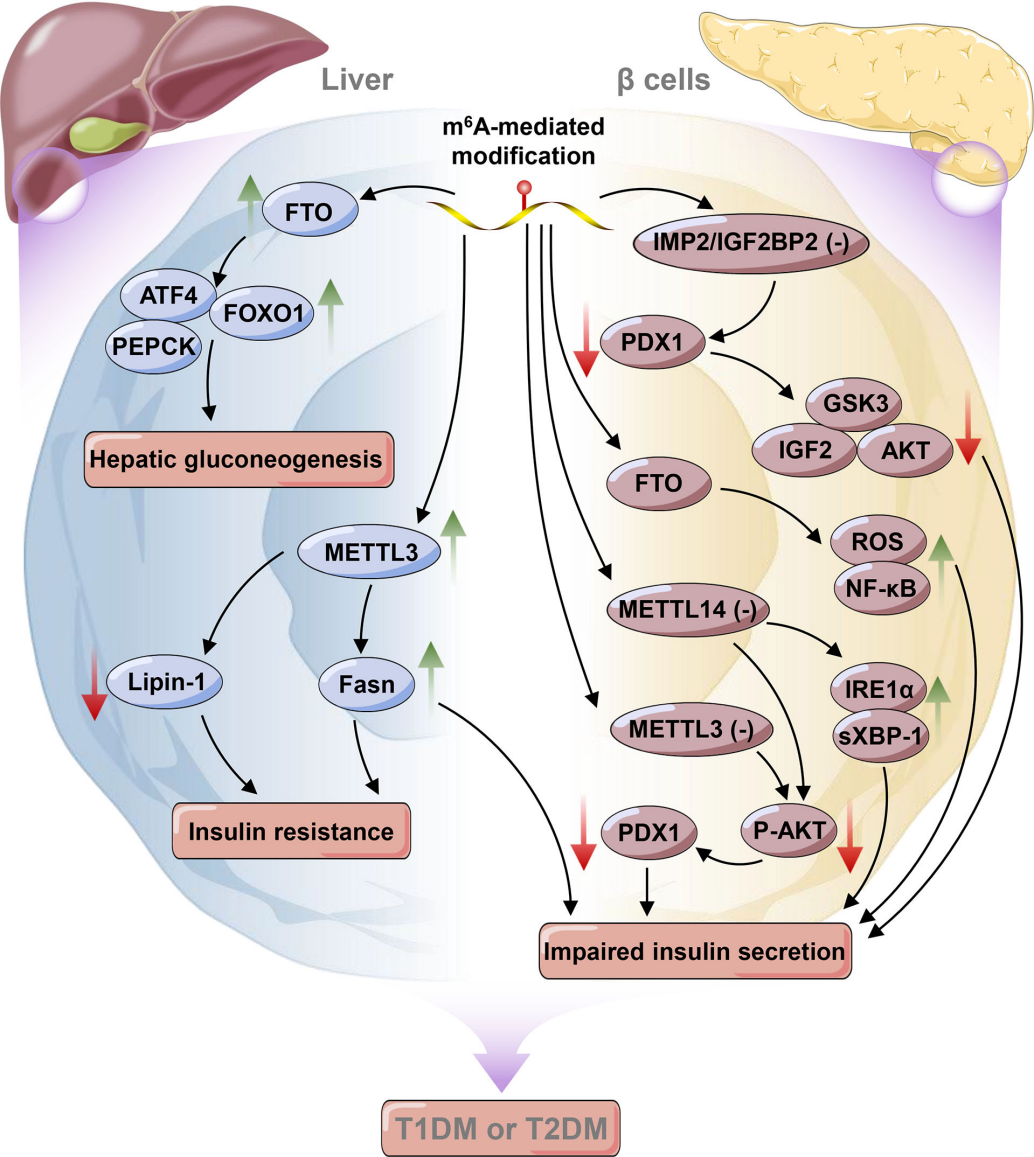
Epitranscriptomic regulatory mechanisms in liver and pancreatic β cells in diabetes mellitus. The green arrow indicates an increase in the change, whereas the red arrow indicates a decrease in the change. *METTL3*, Methyltransferase-like 3; *FTO*, Fat mass and obesity-associated protein; *PEPCK*, Phosphoenolpyruvate arboxynase; *FOXO1*, Forkhead box O1; *ATF4*, Activating transcription factor 4; *Fasn*, Fatty acid synthase; *PEPCK*, Phosphoenolpyruvate carboxynase; *IGF2*, Insulin-like growth factor 2; *IMP2/IGF2BP2*, IGF2 mRNA binding protein 2; *PDX1*, Pancreatic duodenal homeobox 1; *GSK3*, Glycogen synthase kinase-3; *ROS*, Reactive oxygen species; *IRE1α*, Inositol-requiring enzyme 1 alpha; *XBP-1*, X-box binding protein spliced.

Fan et al. found that a lower level of m^6^A was related to increased *FTO* expression instead of *ALKBH5* expression in T2DM patients (108). However, accumulating evidence has demonstrated that both FTO and ALKBH5, two of the most widely known m^6^A demethylases, were important for regulating m^6^A levels despite their different mechanisms of demethylation (30, 39, 50, 123, 124). Bornaque et al. reported increased level of *Alkbh5* mRNA and decreased m^6^A methylation under high glucose condition in pancreatic β cell line (Min6 cells) (50). It is recognized that obesity is the one of the risk factors for the development of IR resulting in T2DM (125). *FTO* has been reported as an obesity-risk gene. Silencing of *Fto* could lead to a decrease in adipose tissue and body mass index, which protected from obesity and metabolic syndrome in mice (125, 126). Mizuno et al. showed that glucose and insulin could keep glucose homeostasis by suppressing the expressions of hepatic *Fto* and gluconeogenic genes, such as phosphoenolpyruvate carboxynase (*Pepck*) and glucose-6-phosphatase (*G6pase*) in liver (127). Furthermore, FTO participates in glucose metabolism in both m^6^A-dependent and nonm^6^A-dependent manners (26). On the one hand, FTO modulates glucose metabolism by affecting forkhead box O1 (FOXO1), activating transcription factor 4 (ATF4), and glucose-6-phosphatase catalytic subunit (G6PC) expression in a m^6^A-dependent manner in response to external stimulation or disease conditions (128, 129). On the other hand, FTO affects glucose metabolism by regulating signal transducers of adipocyte and transcription activator 3 (STAT3), cAMP responsive element binding protein 1 (CREB1), CCAAT/enhancer‐binding protein‐beta (C/EBP‐β), and G6PC expression by a nonm^6^A-dependent pathway under normal conditions (130, 131). Additionally, in pancreatic β cells, overexpression of *Fto* accelerated ROS production and NF-κB activation and consequently inhibited insulin secretion (132) (**Figure 2**). Interestingly, variants in the FTO gene, e.g., rs9939609 and rs1421085, were also reported to be associated with IR and obesity (133, 134). However, some variants associated with BMI (such as rs9930506) in the *FTO* locus are correlated with increased *IRX3* expression rather than *FTO* expression (135).

**Figure 3:**
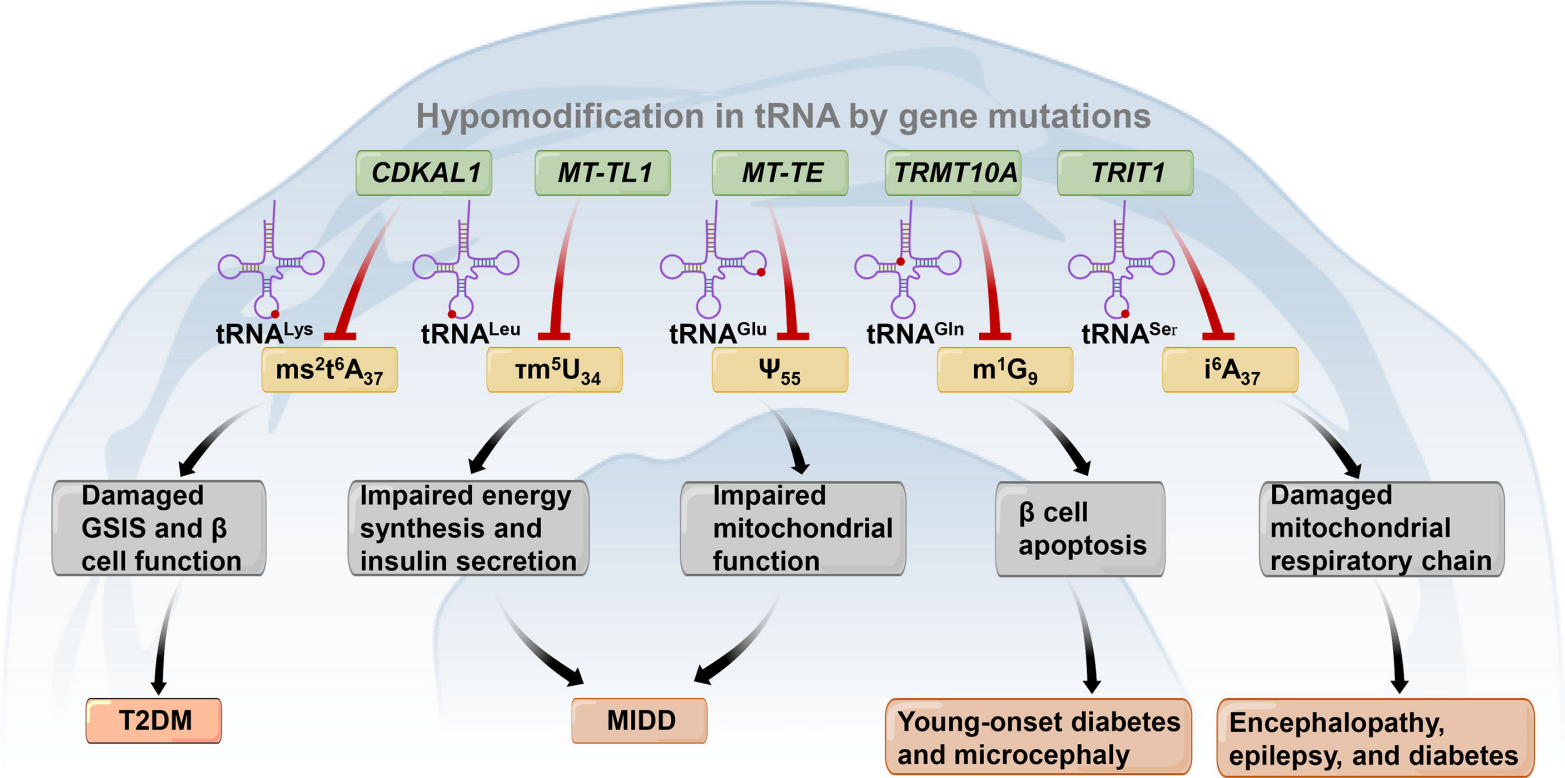
Gene mutations (shown in the blue box) lead to tRNA hypomodifications (shown in the yellow box) associated with diabetes and other metabolic diseases. *CDKAL1*, *CDK5* regulatory subunit associated protein 1-like 1; *TRMT10A*, tRNA methyltransferase 10 homolog A; *TRIT1*, tRNA isopentenyltransferase 1; *ms^2^t^6^A_37_*, 2-methylthio-N6-threonylcarbamoyladenosine at adenosine at position 37 of tRNA^Lys^; τm^5^U_34_, 5-taurinomethyluridine at uridine at position 34 of tRNA^Leu^; *Ψ_55_*, Pseudouridine modification at position 55 of tRNA^Glu^; *m^1^G_9_*, *N^1^*-methylguanine at guanosine at position 9 of tRNA^Gln^. *GSIS*, Glucose-stimulated insulin secretion; *T2DM*, Type 2 diabetes mellitus; *MIDD*, Maternally inherited diabetes and deafness. .

Among all RNAs, tRNAs (both cytosolic and mitochondrial (mt)-tRNAs) experience the most extensive posttranscriptional modifications, with more than 80 chemical modifications on tRNAs manipulated by specific enzymes (136, 137). It has been demonstrated that disorders in tRNA biology are involved in the pathogenesis of many disease conditions, such as cancer and metabolic diseases (137–139). Recently, the crosstalk between abnormal tRNA modifications and pancreatic β-cell function and diabetes has attracted much attention (**Figure 3**). For example, it has been demonstrated that the knockdown of *CDK5* regulatory subunit associated protein 1-like 1 (*CDKAL1*) can reduce CDKAL1-mediated ms^2^t^6^A37 tRNA^Lys (UUU)^ modification and damage GSIS. Thus, it can lead to impaired β-cell function and increased risk for T2DM (140–142). Two kinds of mitochondrial DNA mutations have been discovered to cause maternally inherited diabetes and deafness (MIDD) and are associated with the tRNA epitranscriptome. These mutations are the heterogeneous m.3243A>G mutation and the homoplasmic m.14692A>G mutation. The m.3243A>G mutation can lower mt-tRNA^Leu (UUR)^ 5-taurinomethyluridine modification at position 34 and result in impaired energy synthesis and insulin secretion (138, 143). The m.14692A>G mutation causes the loss of Ψ modification in position 55 (Ψ_55_) of mt-tRNA^Glu^. This leads to the degradation of tRNA^Glu^, subsequently impairing mitochondrial function (144). tRNA methyltransferase 10 homolog A (*TRMT10A*), a tRNA modification enzyme, can methylate guanine at position 9 (m^1^G^9^) in several tRNAs, as previously identified (137, 145). Mutations in *TRMT10A* are recognized as contributors to young-onset diabetes and microcephaly (146–148). A study conducted in clonal human β-cells revealed that the silencing of *TRMT10A* could reduce m^1^G^9^ modification in TRMT10A substrates, including tRNA^Gln (UUG/CUG)^, thus resulting in pancreatic β-cell apoptosis (149, 150). Mutations in *TRIT1* encoding tRNA isopentenyltransferase 1 cause a reduction in i^6^A^37^ modifications in mt-tRNA^Ser (UCN)^ and cytoplasmic tRNA^Ser (UGA)^ and further impair the mitochondrial respiratory chain (138, 151). Consequently, a previous study reported that the homozygous c.968G>A mutation in *TRIT1* could result in encephalopathy, epilepsy, and diabetes (138, 151, 152). These results highlight the vital importance of the tRNA epitranscriptome in β-cell function. These findings may uncover a novel perspective of β-cell death and provide new therapeutic targets for diabetes.

Moreover, in consideration of the crucial relationships between mitochondrial dysfunction, IR and T2DM (153–155), it is also interesting to look at the intersection of the mitochondrion and epitranscriptome without genetic mutation in T2DM. Recently, METTL8, another RNA methyltransferase, was found to promote the 3-methyl-cytidine (m^3^C) methylation of mt-tRNA^Ser/Thr (UCN)^ at position C_32_ (156). Schöller et al. further offered proof that a reduction in METTL8-dependent m^3^C_32_ methylation modifications might inhibit the activities of the components of complex I (mainly ND6 and ND1) and therefore influence the activity of the mitochondrial respiratory chain (157). Moreover, m^1^A modifications on mRNA lead to translational repression due to its destructive effect on Watson-Crick base pairing. This modification has been reported be catalyzed by TRMT61B in the cytosol and TRMT10C in the mitochondrion (158, 159). It is interesting that TRMT10C could facilitate m^1^A methylation in mitochondrial ND5 mRNA, albeit the latter exhibited tissue-specific expression (159). These results suggest that posttranscriptional modifications of RNA in mitochondria play a key role in T2DM and may in part explain mitochondrial dysfunction in this pathological condition. However, the identification of the mitochondrial epitranscriptome in diabetes is still in its infancy, and further studies are warranted.

## The Role of Epitranscriptomics in Diabetic Cardiomyopathy

Diabetic cardiomyopathy (DCM) is a severe complication of diabetes, which is the major cause of mortality in patients with diabetes (160). Over 70% of individuals with diabetes will develop some form of heart disease, such as acute coronary syndrome, during their lifespan (161). It is estimated that 12% of patients with diabetes eventually develop severe heart failure (HF) and often die due to DCM (162, 163). Recent studies demonstrated that diabetes could increase the risk of HF 2-3 times and increase the death rate of HF by 2.5 times in individuals with diabetes compared with control individuals (164). DCM is characterized by myocardium fibrosis, cardiac remodeling and cardiac dysfunction (e.g., contractile dysfunction and reduced ejection fraction) (165). Numerous studies have indicated that the pathogenesis of DCM is complex and multifactorial. It includes the following processes: (1) the overproduction of advanced glycation end-products (AGEs), resulting in extracellular matrix (ECM) accumulation and cardiac fibrosis; (2) excess oxidative stress activating the NF-κB pathway, subsequently leading to myocardial fibrosis; (3) activation of various inflammatory cytokines, activating the proapoptotic signaling pathway and damaging cardiac function; (4) suppression of the insulin signaling pathway, promoting autophagy and apoptosis in myocardial cells; and (5) disturbances in calcium handling, contributing to contractile dysfunction (166, 167).

Previous studies have shown that epigenetic modifications, such as DNA methylation modification, histone modifications, and posttranscriptional RNA regulation (e.g., microRNAs and lncRNAs), play crucial roles in the complexity of the pathogenetic mechanisms underlying cardiovascular disease (CVD) (168, 169). In particular, it has been reported that m^6^A modification extensively participates in various CVDs, such as atherosclerosis (AS), cardiac hypertrophy and HF (12, 30). For instance, Mathiyalagan et al. found decreased FTO expression, cardiomyocyte contractile dysfunction and increased m^6^A RNA modification in failed hearts (170). Overexpression of *Fto* lowered m^6^A levels and improved cardiac contractile function in ischemia-induced HF. It also promoted angiogenesis and inhibited fibrosis in mice with myocardial infarction (MI) (170). In both *in vivo* and *in vitro* studies, increased METTL3 led to m^6^A RNA hypermethylation. It thus enhanced nucleotide-binding domain (NBD) and leucine-rich repeat (LRR) pyrin-domain containing protein 1 (NLRP1) expression and suppressed Kruppel-like Factor 4 (KLF4) expression in a YTHDF1- and YTHDF2-regulated way, respectively (171). These factors could affect atherogenic inflammatory cascades, such as NF-κB p65 Ser^536^ phosphorylation in the vascular endothelium, which ultimately impacts the initiation of atherosclerosis (171). Similarly, Xu et al. discovered that YTHDF2 could hinder cardiac hypertrophy by identifying the m^6^A sites on Myh7 (beta-myosin heavy chain, a marker of cardiac hypertrophy) mRNA and accelerating its degradation (172). Moreover, RNA editing, such as A-to-I editing, has been reported to modulate vascular contraction, arterial remodeling and diastolic blood pressure by editing the actin crosslinking protein filamin A (FLNA) mRNA (173). Nevertheless, exploration of the influence of RNA modifications on the development and progression of DCM is at an early age.

In a study conducted in mice with DCM, researchers identified 984 differentially m^6^A-modified transcripts (including *Mef2a*, *Bcl2l2*, *Slc25a33*, *Cd36*, and *Klf15*), which were closely related to myocardial fibrosis, cardiac hypertrophy, and energy metabolism in the myocardium (174). Interestingly, the AGEs-RAGE pathway has also been demonstrated to participate in DCM pathogenesis by Kyoto Encyclopedia of Genes and Genomes (KEGG) Pathway analysis and protein interaction network analysis of differentially methylated-RNAs in DCM samples. The level of total m^6^A was higher in DCM patients than in normal control individuals. Although several writers and erasers (such as METTL3, METTL14, and ALKBH5) of m^6^A modification were detected, the FTO levels were decreased in DCM. This may contribute to cardiac dysfunction by promoting cardiac fibrosis and cardiomyocyte hypertrophy (174). Thus, m^6^A-dependent epitranscriptomic modifications may be a promising therapeutic approach for DCM.

Pyroptosis is a type of genetically encoded proinflammatory cell death that is different from classic apoptosis. It has been recognized as an essential factor in the pathogenesis of cardiomyopathy and diabetes, especially in DCM (175, 176). For example, a high level of glucose could induce cardiac cell injury and pyroptosis by activating the NLRP3 inflammasome, which is a key regulator of pyroptosis (177). It is worth noting that not only hyperglycemia but also insulin resistance and hyperinsulinemia can lead to the overproduction of ROS and pyroptosis in DCM (6, 176, 178). Xie et al. discovered that chemerin, G-protein-coupled chemokine-like receptor 1 (CMKLR1) and NLRP3 were overexpressed in rats with DCM. They also found that the chemerin/CMKLR1 pathway could activate inflammation and induce pyroptosis in an NLRP3 inflammasome-mediated way, thereby resulting in DCM (179). Accumulating evidence indicates that changes in m^6^A levels can dynamically influence multiple biological processes, such as cell apoptosis, cell proliferation and the inflammatory response (180, 181). Indeed, a recent study found a reduction in METTL14 levels and an increase in the levels of pyroptosis-related markers, including caspase-1, the N-terminus of GSDMD (GSDMD-N), and NLRP3, in both rats with DCM and high glucose (HG)-treated cardiomyocytes (182). Further investigation revealed that METTL14 levels were negatively correlated with terminal differentiation-induced noncoding RNA (TINCR) levels and that the former could downregulate *TINCR* expression by enhancing m^6^A methylated sites in *TINCR* in a YTHDF2-dependent manner. Further study revealed that METTL14 could suppress the expression of lncRNA TINCR and NLRP3, thereby inhibiting pyroptosis and DCM (182). Collectively, these results provide a new perspective on the epigenetic mechanism of pyroptosis in DCM pathologies and suggest that the METTL14-TINCR-NLRP3 axis might be a potential therapeutic target for DCM (182).

Taken together, studies of RNA modifications in diabetic cardiomyopathy are currently lacking. Thus, as new technologies and methods are being developed, more studies on the alterations in levels of epitranscriptomic modifications (e.g., m^6^A and m^5^C, and their regulatory enzymes) in DCM are needed. These investigations could offer novel insights into the physiopathologic mechanisms of DCM and may provide therapeutic targets for this disease given its reversible property.

## The Role of Epitranscriptomics in Diabetic Nephropathy

Diabetic nephropathy (DN), namely, diabetic kidney disease (DKD), is known to be a prevalent microvascular complication of diabetes mellitus (7, 90). It was estimated that more than 40% of individuals with T1DM or T2DM will suffer from DN in their lifespan, Moreover, half of these patients could progress to end-stage renal disease (ESRD), which would significantly increase the mortality rate of patients with diabetes (5, 183, 184). DN is characterized by glomerulosclerosis, epithelial-mesenchymal transition (EMT) of tubular cells, hypertrophy and proliferation of mesangial cells, apoptosis of podocytes, renal vascular disease and so on (185, 186). The pathogenesis of DKD is complex and involves genetic and environmental factors. In recent years, epigenetic regulation, especially RNA modifications, has played a key role in the initiation and progression of DN through diverse mechanisms (7).

For example, m^6^A and ψ were thought to be linked to DN several years ago (187, 188). In a more recent prospective cohort study, Niewczas et al. reported that the level of ψ in serum from patients with diabetes with renal dysfunction was related to damage to renal function and the risk of ESRD (189). Further cross-sectional studies were performed in four groups and revealed increases in m^6^A/C and pseU/U in the DN group. However, the level of I/C was dynamic among the groups. No significant difference in the value of 5-mdC/C and 5-mC/C was observed between the DN group and normal control group (90). Collectively, these candidate metabolites associated with epitranscriptomics may serve as potential biomarkers for the clinical diagnosis of DN and help explain the pathogenesis of DKD. In recent years, epigenetic research has further evaluated DN at the molecular level (190–193).

Klotho, an antiaging gene, has been identified to exert a protective role in DN by alleviating renal tubular and glomerular injury (194, 195). Despite epigenetic modifications, such as DNA methylation and noncoding RNA, METTL14-mediated hypermethylation modification exists in *α-klotho* mRNA and suppresses its expression (190). Li et al. found that the level of METTL14 was positively associated with the levels of ROS, IL-6 and TNF-α and apoptosis in human renal glomerular endothelial cells. Moreover, METTL14 could trigger glomerular endothelial cell damage and the inflammatory response in diabetic mice by inhibiting *α-klotho* expression in a m^6^A-mediated pathway (190).

Podocytes are a critical cell type in the glomerulus that play an essential role in the progression of DKD (196). Elevated expression of *METTL14* was observed both in renal biopsy samples derived from patients with DN and in cultured human adriamycin (ADR)- or AGE-treated podocytes (191). Furthermore, specific *Mettl14* deletion in podocytes *in vitro* could upregulate *Sirt1* expression in a m^6^A-dependent manner. Thus, it could promote autophagy, ameliorate apoptosis, and ultimately improve podocyte injury and glomerular dysfunction (191). Similarly, as another writer enzyme of m^6^A, METTL3 was also shown to be a negative factor in the pathophysiology of podocytes (192). In mouse models of T1DM and T2DM, the level of m^6^A modification in the kidney was markedly increased. Moreover, in renal biopsies from patients with DKD, the expression of METTL3 in podocytes was upregulated. Further study identified that METTL3-modulated m^6^A modification of tissue inhibitor of metalloproteinases 2 (TIMP2) mRNA could promote *TIMP2* expression through the IGF2BP2/Notch3/4 signaling pathway. This could aggravate the inflammatory response, podocyte damage and apoptosis, leading to diabetic nephropathy (192). Considering that posttranscriptional regulation has been considered a fundamental mechanism of podocyte injury in the development and progression of DN, these chemical modifications of RNAs and their related enzymes may be therapeutic targets for this disease. Indeed, a recent study demonstrated that the total flavones of *Abelmoschus manihot* (TFA), which is a kind of Chinese patented medicine extracted from *Abelmoschus manihot*, could improve injury and pyroptosis in HG-treated podocytes. Mechanistically, Liu et al. further demonstrated that TFA exerted this protective effect by activating the PTEN/PI3K/Akt pathway and NLRP3 inflammasome, which are modulated by m^6^A modifications in a METTL3-mediated manner (197).

As mentioned above, EMT of renal tubular cells is one of the classical pathological mechanisms of DKD. It was reported that the overexpression of *METTL14* could suppress EMT in HG-treated human renal proximal tubular cells (HK2) by increasing *PTEN* expression but inhibiting histone deacetylase 5 (*HDAC5*) and *TGF-β1* expression by regulating the PI3K/Akt pathway (193). These results indicate that inhibition of HDAC could alleviate some manifestations of DKD, such as EMT, fibrosis, and albuminuria (198, 199).

## The Role of Epitranscriptomics in Diabetic Retinopathy

Diabetic retinopathy (DR) is a well-known microvascular complication of diabetes and a main reason for visual impairment and blindness in patients with diabetes (200, 201). The pathological mechanisms are complex and include retinal microvascular remodeling, vascular endothelial dysfunction, progressive fibrosis, pericyte dysfunction, neurodegeneration and so on (201, 202). Hyperglycemia-induced processes, such as inflammation, oxidative stress, activation of protein kinase C (PKC) and the renin-angiotensin system (RAS), are considered important factors involved in the pathophysiology of DR (4, 203). Of note, retinal angiogenesis, usually driven by hypoxia, is one of the most vital effects responsible for the pathogenesis and progression of DR (4, 203, 204). In response to hypoxic stimuli, increased levels of m^6^A modification and METTL3 were observed in the retinas of a mouse model as well as endothelial cells (204). Furthermore, METTL3-mediated m^6^A modification aggravates angiogenesis by interacting with YTHDF1 and promoting the expression of genes (*LRP6* and *DVL1*) that take part in the Wnt signaling pathway. This pathway has been demonstrated to undergo significant changes in DR conditions and play a key role in the modulation of retinal homeostasis (204, 205). Interestingly, overexpression of *METTL3* suppressed the EMT of human retinal pigment epithelial (RPE) cells and delayed the progression of proliferative vitreoretinopathy by modulating the Wnt/β-catenin signaling pathway (206).

Although m^6^A RNA modifications and their associated enzymes have been reported to participate in many possible mechanisms of DR, such as inflammation and oxidative stress, their roles in DR remain elusive. Compared to normal control individuals, the expression levels of *METTL3* and *miR-25-3p* were lower in diabetic patients, which is consistent with the results observed in HG-induced RPE cells. An *in vitro* study showed that the overexpression of *METTL3* could upregulate *miR-25-3p*, downregulate *PTEN* and elevate phosphorylated Akt levels in HG-treated RPE cells. These effects alleviated RPE cell pyroptosis and apoptosis under high glucose stress and attenuated the deterioration of DR (207). However, STAT3 signaling was shown to promote the expression of *miR-25* in response to oxidative stress, subsequently resulting in RPE degeneration (208). More research on the role of miR-25 in different pathological mechanisms of DR is necessary. Additionally, microarray analysis of retinal tissues derived from streptozotocin-induced mice with DR revealed that the expression of lysine acetyltransferase 1 (KAT1) was markedly downregulated. In two types of retinal cells treated with high glucose medium, KAT1 promoted YTHDF2-mediated integrin β1 (ITGB1) mRNA degradation in a m^6^A-dependent manner by regulating the FAK/PI3K/AKT axis and thereby improving DR (209). Pericytes are mysterious cells that surround the endothelial cells (ECs) of capillaries. They are involved in microvascular remodeling, which results in diseases in several organs, such as the kidney and retina (201, 202, 210). Pericyte dysfunction has been recognized as the primary initiator of vascular impairment in the pathophysiology of DR (201). A recent study identified increased levels of m^6^A RNA modification and *METTL3* mRNA expression in pericytes and retinas from mice with DR (202). Further study demonstrated that METTL3-modulated m^6^A modification aggravated pericyte dysfunction by suppressing the PKC-η/FAT4/PDGFRA signaling pathway in a YTHDF2-mediated manner (202). Dysregulation of microglia (tissue-specific macrophages in the retina) polarization was considered to be one of the primary pathogenesis of DR (211, 212). Indeed, a higher rate of M1 inflammatory type but lower rate of M2 anti-inflammatory type were observed in both diabetic rats and BV2 cells (a type of mouse microglia cell line) treated with glucose (213). High glucose repressed expression of *Alkbh5* in microglia, which in turn contributed to lower *A20* (also known as tumor necrosis factor-a induced protein 3 (TNFAIP3)) expression and increased M1 polarization; these changes were the results of higher m^6^A modification level and faster degradation rate of *A20* mRNA, which ultimately contributed to the initiation and development of DR (213). Overall, epigenetic regulation of these targets may help control and delay the development and progression of DR, which provides new insights into the pathogenesis and therapy of this disease.

Moreover, upregulation of *METTL3* was found in lens epithelial cells derived from patients with diabetes with cataracts. In HG-induced human lens epithelial cells (HLECs), the level of m^6^A RNA modification was increased, and the expression of *METTL3* mRNA was upregulated. These effects aggravate HLEC apoptosis by governing the 3’ UTR of intercellular adhesion molecule-1 (ICAM-1) (214). Hence, despite the findings in DR, more research focusing on other diabetic-related ocular manifestations, such as glaucoma and ischemic optic neuropathy, is needed in the future.

## The Role of Epitranscriptomics in Diabetic Wounds

Chronic nonhealing wounds are a major threat to patients with diabetes since they can lead to limb amputation (215). Diabetic foot ulcer (DFU), is a wound underneath the ankle that usually results from foot deformities, lower extremity vascular disease and diabetic peripheral neuropathy (216, 217). DFU is one of the most serious complications of diabetes mellitus and can result in high rates of limb loss and disability. Approximately 4–10% of patients with diabetes encounter foot ulcers in their lives, and one diabetic individual loses his or her lower limb due to amputation surgery every 30 seconds worldwide (3, 218). Since DFUs also pose huge threats to personal mental health and the domestic and social economy, it is of great importance to promote wound healing for the treatment of this diabetes-related complication. The etiology of DFUs is multifactorial, including an impaired inflammatory response, reepithelialization, granulation formation, angiogenesis and others, and these processes can be affected by genes, the environment, and nutrition (215, 219). Substantial evidence has demonstrated that epigenetic regulatory mechanisms (such as histone modifications and DNA methylation) play a pivotal role in epidermal homeostasis and participate in the processes of wound healing (215, 220).

It was reported that the expressions of sequestosome 1 (*SQSTM1*)/*p62*, a receptor that regulates autophagy, and the m^6^A reader protein YTHDC1, were greatly decreased in both HG-treated human keratinocyte cells and in the epidermis derived from diabetic mice and patients with diabetes. Moreover, high glucose led to reduced m^6^A level in keratinocytes (218). Furthermore, Liang et at. demonstrated that YTHDC1 could facilitate the instability of SQSTM1 mRNA and autophagy in HG-treated keratinocytes by interacting with ELAV-like RNA binding protein 1 (ELAVL1)/HuR in the progression of wound repair (218). A vast number of studies have demonstrated that keratinocyte dysfunction results in defective epithelialization and contributes to delayed wound healing in DFUs (220, 221). In recent years, stem cell therapy has emerged as a promising treatment for wound repair in diabetes (219). Adipose-derived mesenchymal stem cells (ADSCs) have been suggested to improve wound healing in DFUs and facilitate lymphangiogenesis in previous studies (222, 223). Recent research showed that ADSCs could accelerate vascular endothelial growth factor C (*VEGF-C*) expression through the METTL3/IGF2BP2-m^6^A pathway and promote lymphangiogenesis. Thus, they improved the delayed wound repair in mice with DFU (217). This provides a new potential mechanism for ADSCs involved in the regulation of wound healing and confirms their crucial role in promising therapies for DFU.

However, studies focusing on the relationships between RNA modifications (such as m^6^A) and wound repair in DFUs are very limited. It is recognized that epidermal progenitor cells, which exist in the basal layer of the epidermis, play a crucial role in wound healing and the self-renewal of mammalian skin (224). Transcript analysis of mouse skin epithelial progenitors indicated that transcripts that participate in hair follicle morphogenesis were almost hypermethylated by m^6^A (225). Losses of m^6^A and METTL3 expression contribute to severe defects in hair follicle morphogenesis by regulating several possible signaling pathways, such as NOTCH signaling, BMP signaling, SHH signaling and especially Wnt signaling (225, 226). Another study found that ablation of *Mettl14* could suppress the interaction between Pvt1 and MYC in a m^6^A-dependent manner, thereby damaging epidermal stemness and wound repair in skin (224, 226). Collectively, the relationship between the epitranscriptome and epithelial homeostasis, especially wound repair in DFU, deserves more attention in the future. Additionally, it is particularly important to help understand the molecular basis of epigenetic regulation, which might provide a new perspective for the pathogenesis of diabetic wound healing and the discovery of new therapeutic targets for DFU.

## The Role of Epitranscriptomics in Diabetic Neuropathy

Diabetic neuropathy is a prominent and progressive complication of DM that affects more than 50% of individuals with diabetes and lowers their quality of life (2, 227). The manifestations of neuropathies secondary to diabetes can present as diabetic autonomic neuropathy (DAN) (such as diabetic cystopathy), uremic neuropathy (UN) and diabetic peripheral neuropathy (DPN); of these, the most prevalent is distal symmetric polyneuropathy (DSPN) (2, 228, 229). Previous studies have shown that the occurrence and development of diabetic neuropathy are related to many factors, such as the course of diabetes, control of blood glucose, obesity, insulin resistance and chronic low-grade inflammation. These factors are implicated in various mechanisms, such as overproduction of ROS and AGEs, excessive polyol pathway flux, neurovascular impairments, and perturbations of immunomodulation (2, 230). In addition, gene polymorphisms and epigenomic regulation, such as DNA methylation and posttranscriptional histone modifications, have been shown to participate in diabetic neuropathy in recent studies (230).

Regarding RNA modifications, a growing amount of evidence has demonstrated that epitranscriptomics is involved in neurodevelopmental as well as neurodegenerative diseases in recent years (231–234). For example, in a mouse model with muscle atrophy, denervation resulted in decreased levels of m^6^A and increased levels of the m^6^A demethylase ALKBH5. Overexpression of *Alkbh5* could reduce m^6^A levels and aggravate muscle atrophy after denervation by activating the HDAC4/FoxO3 signaling pathway (235). Furthermore, m^6^A modifications have been regarded as facilitators for epilepsy, Alzheimer’s disease (AD), and Parkinson’s disease (PD) (233, 234). Although the exact mechanisms remain unknown, other RNA modifications, such as m^5^C, pseudouridine, and RNA editing, have also been reported to be associated with Dubowitz-like syndrome, AD, and schizophrenia (231, 232, 234, 236).

Regrettably, links between RNA modifications and diabetic neuropathy have not been well confirmed. A recent study found that inosine could improve sciatic nerve histological structure and function in mice with DPN by regulating the GLO1/AGE/RAGE/NF-κB and TGF-β/PKC/TRPV1/SP axes (227). RNA editing exerts its effects on neurological diseases, as stated in previous studies. Thus, A-to-I editing, which is located in approximately 85% of all premRNAs, may likely play important roles in diabetic neuropathy (234, 237, 238). In short, investigations such as this have guided this hotpot of the current research to explore the potential relationships between RNA modifications and diabetic neuropathy in more extensive studies.

## Specific Small-Molecule Inhibitors Targeting Epitranscriptome

Taking into consideration the dysregulation of the epitranscriptome in diseases such as obesity, diabetes, and cancer, the discovery and design of compounds that may reverse dysregulated epitranscriptome will fire a new and exciting scientific filed. Indeed, some specific activators and inhibitors selectively regulating m^6^A modification have been developed, especially in oncology (239–242). For instance, piperidine and piperazine derivatives can serve as activators of METTL3-METTL14-WTAP complex to promote m^6^A modification on mRNA (243). METTL3 inhibitors, such as UZH1a, have a good potential for the treatment of METTL3-related cancers (244, 245). R-2-hydroxyglutarate, a FTO inhibitor, could suppress the expression of *FTO/PFKP/LDHB* and impair aerobic glycolysis in leukemia (242). Rhein has been demonstrated to function as an inhibitor of FTO by competitively binding the FTO catalytic domain and increasing m^6^A modification in mRNA (246). More FTO inhibitors, such as CHTB, N-CDPCB, meclofenamic acid 2, CS1 and CS2 have shown anticancer effects in existing literature (239, 240). Furthermore, ALK-04, identified as a ALKBH5 inhibitor, together with GVAX/anti-programmed cell death protein 1 immunotherapy, have obvious antitumous effect against melanoma (247). Interestingly, C17 could inhibit PUS7 activity and tRNA pseudouridylation which may impede glioma stem cells growth and thereby suppress glioblastoma tumorigenesis (241, 248). Collectively, these studies highlight the therapeutic potential of compounds targeting epitranscriptomics in cancer therapy.

However, little is known about the effects of these activators and inhibitors in diabetes. And less small-molecule compounds targeting RNA modifying enzymes involved in diabetes and its complications is reported to date. Therefore, it is urgent to design, select and discover potent RNA methyltransferase, demethylase, or RNA-regulating protein inhibitors/activator based on present mechanism researches, which may contribute to the treatment of people with diabetes.

## Conclusions and Future Perspectives

As an epidemic disease worldwide, diabetes mellitus and its complications are still undertreated without efficient therapies in most patients. With the rapid progress of new technologies, such as high-throughput sequencing, epitranscriptomics has recently emerged as a crucial regulatory mechanism involved in both normal physiology and many pathological conditions. Due to its dynamic and reversible nature in response to environmental stimuli, manipulation of RNA modifications might be a promising approach for the treatment and prevention of DM and its related complications.

**Figure 4:**
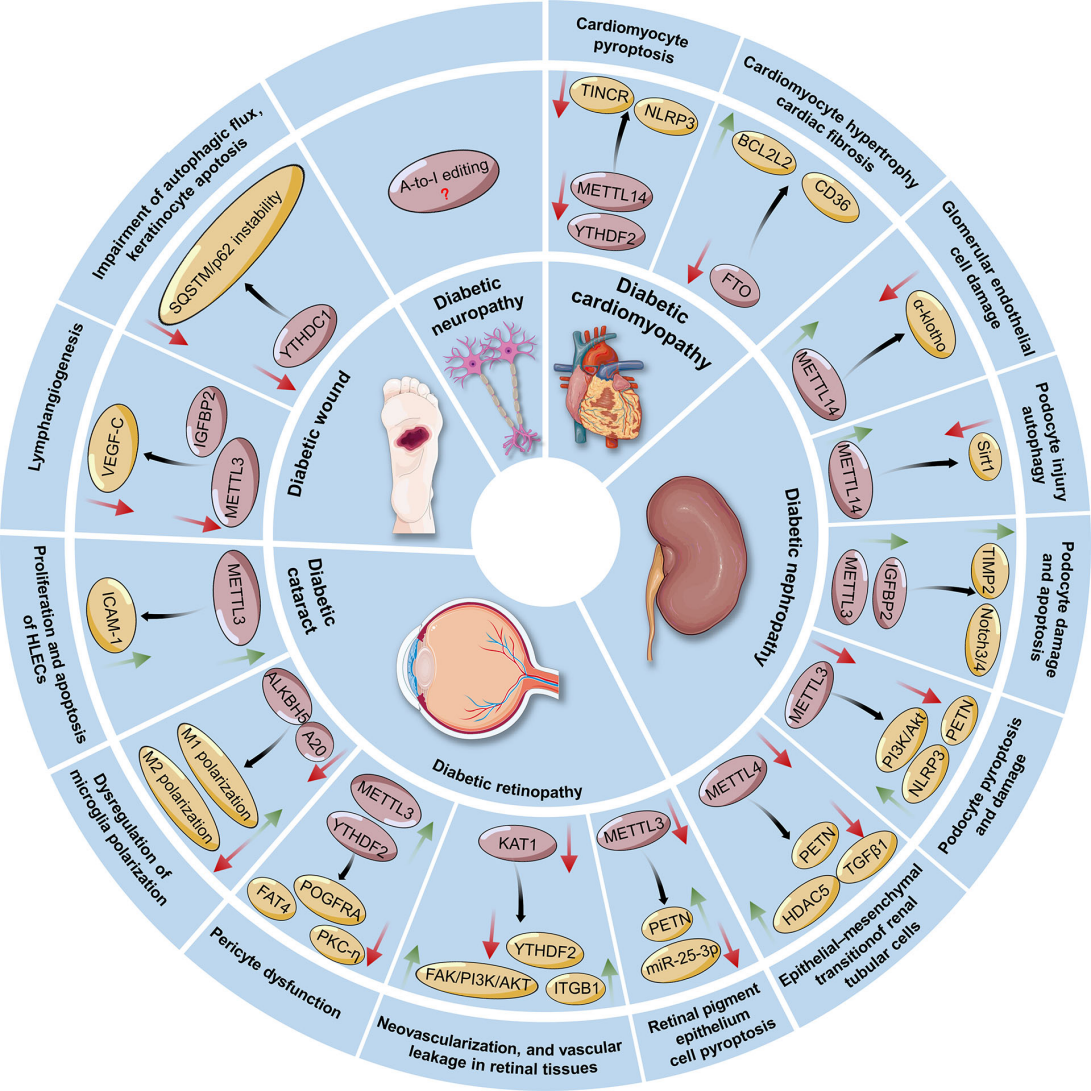
Regulatory roles and possible mechanisms of RNA modifications in the pathogenesis of diabetes-associated complications, including diabetic cardiomyopathy, nephropathy, retinopathy, cataracts, wounds, and neuropathy. The green arrow indicates an increase in the change, whereas the red arrow indicates a decrease in the change. *METTL3/14/16*, Methyltransferase-like 3, 14, 16; *FTO*, Fat mass and obesity-associated protein; *ALKBH5*, ALKB homolog 5; *YTHDF2*, YT521-B homology (YTH) protein families including YTH domain family 2; *YTHDC1*, YTH domain containing 1; TINCR: Terminal differentiation-induced noncoding RNA; *NLRP3*, Nucleotide-binding domain (NBD) and leucine-rich repeat (LRR) pyrin-domain containing protein 3; *TIMP2*, Tissue inhibitor of metalloproteinases 2; *IGF2BPs*, Insulin-like growth factor 2 mRNA-binding proteins; *HDAC5*, Histone deacetylase 5; *ITGB1*, Integrin β1; *KAT1*, Lysine acetyltransferase 1; *ICAM-1*, Intercellular adhesion molecule-1; *SQSTM1*, Sequestosome 1; *VEGF-C*, Vascular endothelial growth factor C. *HLECs*, Human lens epithelial cells; *A20*, Also known as tumor necrosis factor-a induced protein 3 (TNFAIP3); *ALKBH5*, ALKB homolog 5.

However, several gaps in knowledge exist in the understanding of the connection between RNA modification and DM. First, the function of m^6^A and modulating enzymes in diabetes has recently become a topic of interest, and many groups have explored its involvement in various pathophysiologies of diabetes (**Figure 4**). Nonetheless, other RNA modifications, such as m^1^A, m^5^C and m^7^G, are less studied in relation to diabetes. Moreover, studies focusing on the interaction of epitranscriptomics and diabetes-induced complications, such as DCM and diabetic neuropathy, are still insufficient. Hence, future research is warranted to investigate which chemical modifications of RNAs are involved in the pathogenesis of diabetes as well as to determine the exact regulatory and functional mechanisms of these factors that contribute to the occurrence and development of this disease. Second, some RNA modifications are cell type-specific or organ-specific. That is, whether RNA modifications exhibit their protective or destructive effects in response to metabolic or environmental stress depends on the cellular context and specific transcripts involved. To this end, there is a challenge to understand the condition-specific influences of RNA modifications on a specific transcript as well as the precise mechanisms by which RNA modifying enzymes differentially regulate the given transcript into a specific functional protein. Moreover, it is urgent to identify more RNA modification writers, readers, and erasers and determine the specificity of these regulatory proteins. Such findings could be beneficial for understanding the mechanisms of these proteins involved in both physiological homeostasis and pathological conditions. Third, similar to the identification of small molecule inhibitors against DNA methylation and histone modifications, it is of utmost importance to discover small molecules that target RNA modifying enzymes. These may shed light on the exploration of epigenetic regulatory mechanisms underlying diabetes pathogenesis and the identification of “epidrugs” in the era of precision medicine. Collectively, it is essential to explore whether the overexpression or knockout of RNA modifying enzymes (writers, erasers, and readers) can be taken from bench to bedside for diabetes management in future studies.

## Data Availability Statement

The original contributions presented in the study are included in the article/supplementary material. Further inquiries can be directed to the corresponding author.

## Author Contributions

XG contributed to the conception and first draft of the manuscript. ZL contributed to the figures and writing. YY contributed to the revision and editing of the manuscript. All authors reviewed the manuscript and approved the final draft.

## Funding

The work was financially supported by funding from Basic Research Program of Yunnan Province (Kunming Medical University Joint Special Project) (No. 202101AY070001-276), the National Natural Science Foundation of China (No. 82160159), the Key Field R&D Plan Project of Guangdong province (No. 2019B020201002) and the Innovative Team of Precise Prevention and Treatment against Metabolic Diseases of Yunnan University.

## Conflict of Interest

The authors declare that the research was conducted in the absence of any commercial or financial relationships that could be construed as a potential conflict of interest.

The handling editor ZZ declared a shared parent affiliation with the authors XG and YY at the time of review.

## Publisher’s Note

All claims expressed in this article are solely those of the authors and do not necessarily represent those of their affiliated organizations, or those of the publisher, the editors and the reviewers. Any product that may be evaluated in this article, or claim that may be made by its manufacturer, is not guaranteed or endorsed by the publisher.
